# Lack of gastric acidification reduces postprandial energy expenditure and protein digestion but not growth in *Astyanax mexicanus*

**DOI:** 10.1242/jeb.251599

**Published:** 2026-04-30

**Authors:** Patrícia Gomes Ferreira, Hugo Flávio, Jonathan M. Wilson

**Affiliations:** ^1^ Department of Biology, Wilfrid Laurier University, Waterloo, ON, Canada, N2L 3C5; ^2^ Department of Biology, Dalhousie University, Halifax, NS, Canada, B3H 4R2

**Keywords:** Acid-peptic digestion, Crispr-Cas9, Omeprazole, Mineral assimilation

## Abstract

The vertebrate stomach is responsible for the secretion of hydrochloric acid (HCl) and is the first site of protein digestion in the gut. The secretion of HCl occurs through the gastric proton pump, a hydrogen–potassium ATPase (HKA) composed of α and β subunits encoded by the *ATP4A* and *ATP4B* genes, respectively. In the past, the evidence for the role of the gastric acid secretion in nutrient digestion and absorption, growth and postprandial energy metabolism has been gathered using indirect methods such as diet modulation experiments, or the use of proton-pump inhibitors. These methods may introduce confounding factors and lead to erroneous conclusions. With the aim of directly observing the role of the gastric proton pump, we have generated a knockout model using targeted gene editing. Using *atp4a*-null *Astyanax mexicanus*, we examined the growth rate, nitrogen and energy metabolism, and nutrient assimilation in the presence and absence of gastric acidification. Our results show no effect of knockout on growth or appetite, but a significant reduction in post-prandial nitrogen excretion and oxygen consumption (specific dynamic action). Furthermore, *atp4a^−/−^* animals had significantly less body magnesium, calcium, phosphorus and protein, while having more lipid in their carcasses. Importantly, administration of proton-pump inhibitors suppressed growth in both experimental groups, indicating possible off-target effects of these drugs. This study is the first to directly examine the impact of gastric acidification on body composition, growth and metabolism and offers new and targeted evidence on the importance of stomach acidification for gut and digestion homeostasis.

## Teaser

**Summary:** An *Astyanax mexicanus* gastric proton pump knockout model shows that lack of stomach acid impacts postprandial energy expenditure (specific dynamic action) and mineral composition without noticeably impacting growth.

## INTRODUCTION

The capacity for acid-peptic digestion was a major innovation in the evolution of the digestive system that gave rise to the stomach phenotype of vertebrates (Koelz, 1992; Smit, 1968). Acid production is essential for the activation of pepsinogen into the aspartic peptidase pepsin, an important enzyme in protein digestion (Kageyama, 2002; Richter et al., 1998). In addition, acid secretion has been shown to be important for the solubilization of phosphorus (P), and consequently calcium (Ca) fixation in bones, as well as magnesium (Mg) absorption (Sugiura, 2025; Kopic and Geibel 2013). Gastric acid secretion is performed by the highly conserved gastric proton pump (HKA), a heterodimeric enzyme encoded by the *ATP4A* gene (α subunit) and the *ATP4B* gene (β subunit; Shin et al., 2009). In spite of the importance and conservation of acid-peptic digestion in vertebrates, secondary loss is not uncommon (Koelz, 1992; Castro et al., 2014; Kato et al., 2024; Ferreira et al., 2025a).

The secretion of gastric acid into the stomach lumen during digestion is driven by the ATP-dependent movement of hydrogen ions (H^+^) against a large gradient (160 mmol l^−1^) in exchange for potassium cations (K^+^; Kopic et al., 2010; Shin et al., 2009). The intracellular H^+^ (and bicarbonate, HCO_3_^−^) is produced by the hydration of carbon dioxide (CO_2_) catalyzed by cytoplasmic carbonic anhydrase in the oxynticopeptic cells of the gastric glands of fish (CO_2_+H_2_O⇌H_2_CO_3_⇌H^+^+HCO_3_^−^). The apical secretion of H^+^ is complemented with the basolateral secretion of HCO_3_^−^ into the blood (Ferreira et al., 2025b). This in turn generates a transient acid–base imbalance called the postprandial alkaline tide (first described by Jones, 1845, and reviewed by Niv and Fraser, 2002). The alkaline tide has been widely characterized in mammals (Brunton, 1933; Niv and Fraser, 2002), and more recent studies have characterized it in several fish species, from chondrichthyans (Wood et al., 2007) to teleosts (Bucking et al., 2009; Cooper and Wilson, 2008). In fish, the immediate recovery of acid–base homeostasis is achieved through the excretion of HCO_3_^−^ mainly by the gills before HCO_3_^−^ is secreted into the anterior intestine to neutralize the acidic chyme (reviewed by Wood, 2019). This recovery process presumably contributes to the overall energetic cost associated with the maintenance of the gastric proton pump.

Pharmacological inhibition of acid secretion through the use of omeprazole, a proton pump inhibitor (Shin and Sachs, 2008), has been associated with a reduction in growth in Nile tilapia (*Oreochromis niloticus*) as a probable consequence of reduced protein digestibility (Moffatt et al., 2022). The gastric proton pump is essential for the initiation of protein digestion in the stomach (Kageyama, 2002). The pepsinogens, secreted by the oxynticopeptic cell, are activated in the presence of hydrochloric acid (HCl) and are the first peptidases to act on the chyme. The dietary amino acids that are not used in protein synthesis are not stored in the body, unlike lipids and carbohydrates (Chandel, 2021). Thus, after absorption, excess dietary amino acids are catabolized, and fish experience an overall increase in ammonia levels (NH_3_ and NH_4_^+^) in the plasma and consequent increased ammonia excretion into the water through the gills (Bucking et al., 2009; Kaushik and de Oliva Teles, 1985). This increase in ammonia is largely due to the catabolism of digested protein and the metabolism of the gut tissue and liver (Taylor et al., 2010; Rubino et al., 2014). The secretion of gastric acid activates a pH-sensitive response in the anterior intestine that triggers the release of intestinal hormones such as cholecystokinin (CCK; Holstein, 1982; Holmgren and Holmberg, 2005; Volkoff et al., 2005). The secreted CCK binds to CCKb receptors in the pyloric sphincter's smooth muscle and in afferent vagal neurons leading to a decrease in stomach motility and, consequently, to a delay in the rate of gastric emptying in mammals (Smith et al., 1984; Lin and Miller, 1992; Corp et al., 1993; Olsson et al., 1999). Moffatt et al. (2022) showed significantly increased gastric emptying rates in Nile tilapia after omeprazole treatment, supporting the physiological importance of gastric acid in the regulation of gastric transit time. In addition, gastric bypass surgery has been linked to decreased gastrointestinal transit time in mice (Liou et al., 2013).

The energetic costs associated with nutrient ingestion, digestion and assimilation are commonly termed the specific dynamic action (SDA) and translate into a transient, post-prandial elevation of metabolic rate (Chabot et al., 2016). Studies over the past decades have comprehensively examined this phenomenon in various taxa and found that it significantly contributes to an animal's energy budget (Beamis et al., 1975; Fu et al., 2005; Goodrich et al., 2022a,b; Secor, 2009). The SDA response is likely to include a broad set of processes spanning from pre- to post-absorptive phases of digestion. Although several studies have attempted to examine the contribution of the gastric acid digestion to the magnitude of SDA using proton pump inhibitors or diet modulation, the results have been contradictory and species dependent (Andrade et al., 2005; Goodrich et al., 2022a,b; Nørgaard et al., 2016; Secor, 2009). There will be a direct cost of acid secretion because the gastric proton pump is an ATP-dependent process with a stoichiometry of 1 H^+^:1 ATP. The estimates of H^+^ pumped per O_2_ consumed range from 5.0 to 2.3 (Kopic et al., 2010 and Bannister, 1965, respectively; see Goodrich et al., 2022a). However, it is clear that a significant component of SDA is tied to processes that include increased rates of protein synthesis (37.2 mmol ATP or 6.2 mmol O_2_ per gram protein synthesis; Houlihan et al., 1993) and the metabolism of nutrients after intestinal absorption (Brown and Cameron, 1991a,b; Lyndon et al., 1992; Smith and Houlihan, 1995), but a more broad understanding of the contributors to the SDA remains to be fully characterized.

The use of HKA-null animals (which lack gastric acidification) is an excellent tool for clarifying the specific role of gastric acid in growth and to further dissect the components and allocation of post-prandial energy utilization, and illuminate the consequence of the evolutionary loss of the stomach phenotype (Ferreira et al., 2025a). In the present study, we compared energy consumption, body composition, nitrogen excretion, acid–base homeostasis and growth in previously generated knockout (*atp4a*^−/−^) and wild-type (*atp4a^+/+^*) *Astyanax mexicanus* (Ferreira et al., 2025b). The Mexican tetra, *A. mexicanus*, is a small freshwater fish that is emerging as a powerful evo-dev model organism (Swaminathan et al., 2024). The knockout fish are achlorhydric, with circumneutral stomach pH levels, and lower acid-peptic gene (e.g. *atp4b*, *pga*, *pgc*) expression in contrast to wild-type fish, that notably have a post-prandial stomach pH of 3.8 (Ferreira et al., 2025b). We hypothesized that the absence of gastric acidification in *atp4a^−/−^* fish decreases the magnitude of both the SDA (measured by intermittent flow respirometry) and the alkaline tide. Furthermore, we predicted that *atp4a^−/−^* fish would experience impaired protein digestion, reduced growth and faster gastric transit times. An alternative scenario could reflect that the reduction in the energetic cost of digestion would enhance fish growth and food conversion efficiency. To characterize the impacts of gastric acidification on mineral and protein digestion, we analyzed Mg, Ca, P, total protein and lipid content in the carcass. Finally, to assess potential secondary targets of the proton pump inhibitor omeprazole, we included a 2-week omeprazole treatment in our growth trial.

## MATERIALS AND METHODS

### Animals

*Astyanax mexicanus* (De Filippi 1853) (surface morph) were originally obtained from the Tabin lab at Harvard Medical School (Boston, MA, USA). Fish were maintained as described previously in Ferreira et al. (2025b), and kept at 22.5°C in recirculation systems with artificial freshwater – reverse osmosis water adjusted to 700–800 µS cm^−1^ with sea salt (Instant Ocean). All animal experiments were approved by the Animal Care Committee at Wilfrid Laurier University (AUP R22002). The knockout line for *atp4a* was previously characterized by Ferreira et al. (2025b). The mutant animals have a net +16 bp change in exon 11 that results in a premature stop codon 536 aa downstream of the start codon of Atp4a, resulting in a truncated and non-functional protein and consequently the loss of gastric acidification. Henceforth, we refer to wild-type animals as *atp4a^+/+^*, and homozygous knockout animals as *atp4a*^−/−^.

### Growth trials

Homozygous (*atp4a*^−/−^) and wild-type (*atp4a^+/+^*) fish (1.5 months old, mixed sex, *n*=10) were placed in individual containers in a recirculating aquatic rack system supplied with artificial freshwater kept at 22.5°C. The food was carefully weighed on a precision balance. The weighing of the micro-pellets (feed) was reproducible and a pilot feeding trial was conducted beforehand to ensure feasibility. The fish were habituated to the system and feed (Tropical micro pellets, Hikari; see Table S1 for pellet dietary formulation) for 2 weeks prior to starting the trial. Fish were weighed on an analytical balance, after briefly blotting the excess water from their body (knockout 0.0865±0.0342 g; wild-type 0.1155±0.0486 g) at the beginning of the trial and fed a 3% body mass (*M*_b_) daily ration. The fish consumed the entire meal within a few minutes. Recirculation was paused for 15 min before feeding until 1 h post feed. We ensured that the entire meal was consumed before re-starting the system's recirculation. Ration size was adjusted on a weekly basis following re-weighing. After 3 weeks of regular diet feed (Control period), all animals were switched to a diet supplemented with omeprazole to evaluate possible off-target effects of this proton-pump inhibitor (omeprazole period). After 2 weeks on the omeprazole diet, fish were switched back to the regular diet (Hikari, 3% *M*_b_) for 3 weeks (recovery period). Specific growth rates (SGR=*G*) were calculated following Crane et al. (2020), as follows:
(1)

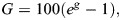

(2)

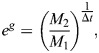
where *g* is the instantaneous growth rate, *e* is the natural log, *M*_1_ and *M*_2_ represent initial and final mass, respectively, and Δ*t* represents the elapsed time between measurements (days). The feed conversion ratios (FCRs) were calculated for each group and trial week by dividing the feed intake (mg) by the mass gain (mg).

#### Omeprazole diet preparation

Omeprazole (Tokyo Chemical Industry) was added to the Hikari pellets in a dose of 25 mg kg^−1^ day^−1^ dissolved in 95% ethanol based on a 3% *M*_b_ ration (Wood et al., 2009; Moffatt et al., 2022). In short, the pellets were air dried to completely evaporate the ethanol, stored at −20°C and used within a week.

### Acid–base fluxes

Homozygous and wild-type animals (*n*=8 per group) were fasted for 48 h prior to the acid-flux measurements. On the morning of the fluxes, the animals were voluntarily fed a 5% *M*_b_ bloodworm meal and transferred to aerated static flux chambers (50 ml). The water samples were collected at 0, 3, 6, 9 and 24 h after transfer. Faeces were also collected from the chambers during the 24h flux period for analysis. The fish were transferred back to their original containers after the 24 h flux period. The titratable alkalinity of chamber water samples was measured following McDonald and Wood (1981) by titrating 10 ml of water to an endpoint of pH 4.3 with an autoburette/titrator system (Radiometer Copenhagen ABU 80 autoburette/TTT 80 titrator), followed by a manual titration to an endpoint of pH 4.0 with 0.01 mol l^−1^ HCl (Sigma-Aldrich). The total ammonia in the water was measured using the salicylate-based colourimetric method following Cooper and Wilson (2008) based on Verdouw et al. (1978). The net fluxes of titratable alkalinity (*J*_TAlk_) and total ammonia (*J*_TAmm_) for each flux period were calculated using the following equation from Cooper and Wilson (2008):
(3)

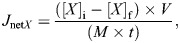
where [*X*]_i_ and [*X*]_f_ are the initial and final values for ammonia (μmol l^−1^ total ammonia nitrogen, TAN) or titratable alkalinity (µEq l^−1^), *V* is the volume of water (l), *M* is mass of the fish (kg) and *t* is the duration of the flux period (h). The difference between *J*_TAlk_ and *J*_TAmm_ (µmol kg^−1^ h^−1^) was used to calculate the net acid–base flux (µEq kg^−1^ h^−1^). The faeces collected during the acid flux measurements were photographed using a Leica M165FC stereomicroscope with a DFC6200 camera. The relative length of the faeces was measured using LASX software, with a minimum of 20 faecal pellets measured per animal (*n*=6 animals per genotype). The faeces were then dried at 60°C for the determination of total nitrogen content.

### Respirometry

The oxygen consumption rate (*Ṁ*_O_2__ in µmol O_2_ g^−1^ h^−1^) was determined using intermittent flow respirometry. Details on the system setup, phase duration and methods of *Ṁ*_O_2__ and SDA calculation are provided in the Supplementary Materials and Methods.

The day before the experiment, the animals were weighed (to the nearest 0.0001 g using an analytical balance) to determine the ration size. The following day, the animals were voluntarily fed (5% *M*_b_ wet bloodworm meal) 15–20 min before being placed inside the respirometers. The respirometers were covered with a black plastic tube to minimize visual stimuli. Measurements of *Ṁ*_O_2__ were initiated immediately after the transfer of each animal to the respirometer. The animals were allowed to remain in the chambers undisturbed for 24 h (pilot trials showed that this time sufficed to capture SDA and standard metabolic rate, SMR). In total, 26 animals were used in these experiments, resulting in 20 post-prandial traces deemed appropriate for SDA estimations. Five animals were excluded because they showed high activity levels throughout the experiment (high noise levels in the measurement points/O_2_), preventing an accurate estimation of SDA. One last animal was excluded because it displayed a very short SDA (approximately 3 h), so it is likely to have vomited the meal. Of the 20 animals used for the analysis, eight were *atp4a^+/+^* (0.40±0.04 g) and 12 were *atp4a^−/−^* (0.35±0.03 g). The *Ṁ*_O_2__ for each cycle was determined using the R package pyroresp (https://github.com/hugomflavio/pyroresp), in R v4.4.1 (https://www.r-project.org/).

### Appetite trial

The appetite levels of knockout and wild-type fish were determined following a modified appetite assay from Aspiras et al. (2015). Animals (*n*=8 per group) were fasted for 48 h and then offered a pre-weighed (∼0.5 g) bloodworm meal. After 24 h, the remaining bloodworms were separated from faecal matter and reweighed. The appetite results were normalized to the condition factor (*K*=100*M*_b_ *L*^−3^, where *M*_b_ is body mass in g and *L* is length in cm; Ricker, 1975; Aspiras et al., 2015).

### Tissue sampling

Fish (*atp4a^−/−^ n*=12, *atp4a^+/+^ n*=8) were fed a bloodworm meal (5% *M*_b_) and euthanized 3 h post feeding with an overdose of tricaine methanesulfonate (1:5000 Syndel, Nanaimo, BC, Canada) buffered with NaHCO_3_ to pH 7. The stomach contents were emptied and weighed to determine stomach emptying. In addition to stomach, the brain and intestine (after removal of the chyme) were also snap-frozen and kept at −80°C until further use. The remaining visceral organs were excised from the body cavity, and the carcasses were weighed, dried at 85°C and weighed again for total water determination.

#### Compositional and element carcass analyses

All dried carcasses, feed and faeces were manually ground using a mortar and pestle into a powder and kept in moisture-free conditions until further analysis. Total lipid content was determined gravimetrically in ground carcasses using established methods (AOAC, 2000; detailed methods can be found in the Supplementary Materials and Methods). The Mg, Ca, Na and P content in the carcasses and feed were quantified through inductively coupled plasma-optical emission spectroscopy (Optima 8000 ICP-OES spectrometer, Perkin Elmer). Twenty milligrams of dried-ground carcass and feed (pellets and bloodworms) samples were weighed and transferred to clean glass culture tubes containing 2 ml of 20% (v:v) nitric acid (HNO_3_). The tubes were heated at 105°C for 30 min and manually agitated every 10 min. After digestion of the samples, the tubes were cooled to room temperature, and the contents were transferred to clean 15 ml capped tubes with the volumes adjusted to 15 ml with ultrapure water. The samples were centrifuged for 5 min (EC Clinical Centrifuge Damon) and transferred to new clean 15 ml tubes. Elemental standards (sodium phosphate monobasic, calcium chloride dihydrate and magnesium chloride hexahydrate) were prepared in a final concentration of 1000 mg l^−1^, following the same protocol as the preparation of the sample, including digestion in HNO_3_.

The total nitrogen content of the dry-ground carcass and faecal samples was determined using a 2400 CHNS analyzer (Perkin Elmer). One to three milligrams of dry sample were analyzed, and acetanilide (71.09% C, 6.71% H, 10.36% N; BDH Organic Analytical Standard) was used as a standard to verify calibration before and during the analysis. Total nitrogen was converted to protein using the standard conversion coefficient of 6.25, and the final values were expressed as a percentage of protein per dry mass (Mariotti et al., 2008).

#### Determination of ammonia content in the chyme and feed

The quantification of ammonia in the chyme and the feed (bloodworm meal) was carried out using a commercial assay (ammonia assay kit AA0100-1KT, Sigma-Aldrich; glutamate dehydrogenase method). *P*_NH_3__ in the chyme was determined using p*K*′ and α NH_3_ values reported for 23°C by Cameron and Heisler (1983), and ionic strength was assumed to be 125 mmol l^−1^ (Rubino et al., 2014). NH_3_ and *P*_NH_3__ concentrations were calculated following Rubino et al. (2014).

#### Gene expression

Total RNA from the brain and intestine samples was extracted using a RNeasy Mini Kit with on-column DNase treatment (Qiagen, Hilden, Germany). Total RNA (1 µg) was converted into cDNA using the High-Capacity cDNA RT kit (Applied Biosystems). Quantitative real-time PCR (qPCR) was used to evaluate gene expression profiles of genes related to growth, gastric evacuation and satiation (Table 1), using the BlasTaq™ 2X qPCR Master Mix (Applied Biological Materials Inc., Richmond, BC, Canada) on a BioRad CFX96 real time system under the following cycle conditions: denaturation 95°C 3 min, and 40 cycles of 95°C for 15 s, 58°C for 30 s and 72°C for 2 s. Melt curve analysis was performed after each run to confirm single band products. Relative expression levels were normalized with a geometric mean using elongation factor (*ef1a*), *18s* and *gapdh* gene expression. Reference gene primer sequences were previously published by Imarazene (2020) (*ef1a* and *18s*) and Ferreira et al. (2025b) (*gapdh*). Relative gene expression quantification was calculated using the 2^−ΔΔCT^ method (Livak and Schmittgen, 2001).

**Table 1.** Primers used in the study, qPCR efficiency values (*E*) and accession numbers

Gene	Forward primer	Reverse primer	*E* (%)	Accession number
*lepa*	CAACGAGATGAGCTGCCGAT	CAGGCCTTCGATGGGCTTAT	103.2	XM_049483678.1
*mc4r*	GGACAGTAATTGACTGCTGCTT	ACGTGGCACCATGTTGTACT	106.1	XM_007232098.4
*insra*	CGAGCCAAAAGCTCCCAATG	GCTTACAGCCATTGTCCGTG	92.8	XM_007236326.4
*npy*	ACGAGGCAGAGGTATGGGAA	ATCACCACATCAACGGGTCG	89.7	XM_049476010.1
*pyy*	GAAAACCCAGGAGACGATGC	CCCTCTGGAGTGGACCTTTT	108.2	XM_022671994.2
*ghra*	GCATTCGACAACTTTGGGGA	ATCCCCACCACACCAAACA	98.9	XM_049485692.1
*ghrb*	CAAGTGCTCTTCAACGTGGA	AGCGACACTCAGTAAAGTCCA	98.1	XM_022675925.2
*cckb*	AAGGTGGAGATGTAGGTGCA	GCTCTCCTTGAACTTGCAGG	95.3	XM_022676228.2

#### Cholecystokinin ELISA

Frozen anterior intestine samples (3 h post-prandial) were homogenized in phosphate-buffered saline (PBS) using a bead homogenizer (Precellys 24; 6500 rpm for 2×10 s; Bertin Technologies SAS, Montigny-le-Bretonneux, France) and centrifuged at 21,100 ***g*** for 15 min at 4°C. A fish-specific Cck ELISA assay was used to measure expression (Cusabio Tech LLC). The total protein concentration of samples was determined using the BCA assay with a BSA standard. Cck is expressed as pg Cck µg^−1^ protein.

### Data analyses

Before we tested for differences between treatment groups, the normality of the data was verified by a Shapiro–Wilk test, followed by a homoscedasticity test. For most datasets, differences between the two groups were tested with a two-tailed independent *t*-test (when data were normally distributed) or a Wilcoxon rank sum test (when data were not normally distributed). For the analysis of the growth and flux data, a two-way repeated-measures ANOVA was used, followed by a Tukey HSD *post hoc* test. For the respirometry data, because variance was unequal between the groups for the four variables of interest (i.e. SMR, SDA duration, SDA net peak and SDA magnitude), differences between the groups were tested using Welsh two-sample *t*-tests. *P*-values lower than 0.05 were considered statistically significant. All statistical analyses were performed in R 4.4.0. Data are presented as means±s.e.m.

## RESULTS

### Lack of gastric acid does not impact growth in *A. mexicanus* but illustrates potential off-target effects of omeprazole

**Fig. 1.**
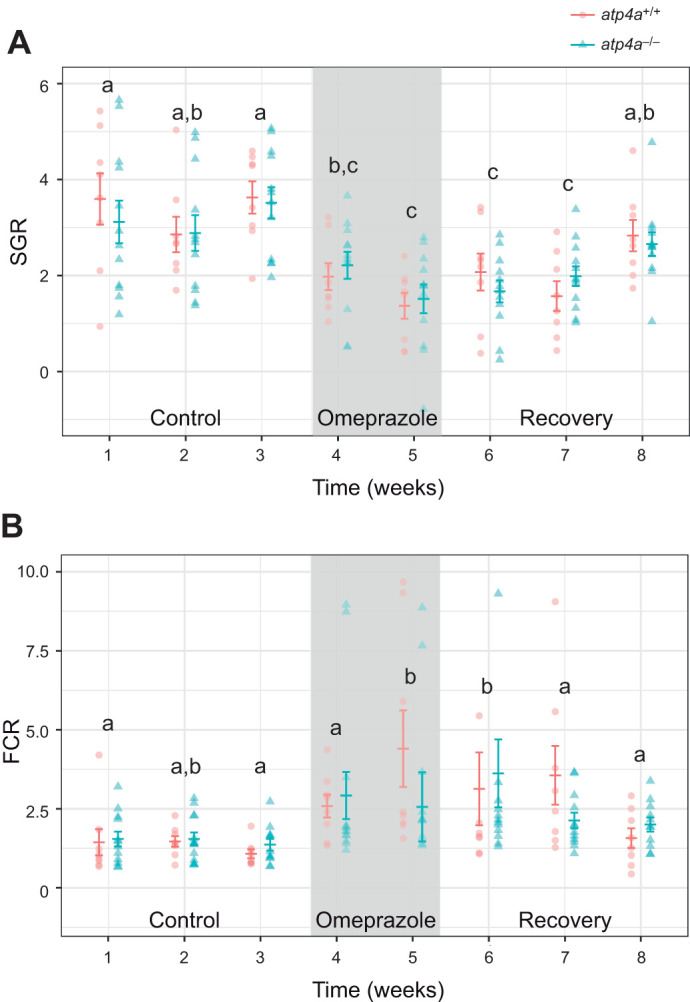
***Astyanax mexicanus* growth trial.** Both *atp4a^+/+^* and *atp4a*^−/−^ animals were fed a 3% body mass (*M*_b_) ration of pellets. In the first 3 weeks, both groups were fed non-treated pellets. In weeks 4 and 5, both groups were fed the same ration size of pellets dosed with omeprazole (25 mg kg^−1^ *M*_b_ day^−1^). Weeks 6–8 were recovery weeks (post-omeprazole), where both groups were fed untreated pellets. Animals were weighed every week throughout the trial period. Specific growth rates (SGR, A) and feed conversion ratios (FCR, B) were calculated over these weekly intervals. Data were analyzed by two-way ANOVA with Tukey *post hoc* test; *n*=8 *atp4a*^+/+^ and *n*=12 *atp4a*^−/−^. There were no significant differences nor interactions between genotypes. Significant differences were noted between trial weeks. The time intervals labelled with different letters are significantly different from each other (*P*<0.05).

The SGRs were calculated weekly during the 8 weeks of the growth trial (Fig. 1A). The fish consumed the entire 3% *M*_b_ meal in less than 3 h. The mass of the two groups was not significantly different from each other at the beginning of the trial (*atp4a^+/+^* 0.0865±0.0342 g, *atp4a^−/−^* 0.1155±0.0486 g, *t*_18_=1.570, *P*=0.134). SGRs were similar between genotypes throughout the 8 weeks (*P*=0.670). There was no interaction between genotype and trial period weeks (control 1–3, omeprazole 1–2 and recovery 1–3: *F*_7,125_=0.506, *P*=0.829). Both groups had a significantly reduced SGR in the second week of omeprazole treatment (∼45.2% reduction). This depression in growth rates was sustained until the last week of the recovery phase (week 8 of the trial), at which time both groups showed recovery to control levels of growth. Although no differences in SGRs were detected between genotypes, the final body mass at the end of the trial indicated that knockout fish were smaller than wild-type fish (*atp4a*^+/+^ 0.3234±0.0217 g, *atp4a*^−/−^ 0.2469±0.0313 g, *t*_18_=2.258, *P*=0.037). The FCR did not differ between genotypes (*P*=0.967) and there was no interaction between genotype and trial period (interaction *F*_7,126_=0.796, *P*=0.592; Fig. 1B). The FCR was significantly higher compared with the control levels in the second week of omeprazole treatment, returning to levels similar to control from week 7 of the trial period.

### Lack of gastric acid reduces branchial ammonia excretion with no discernible impact on acid–base fluxes

**Fig. 2.**
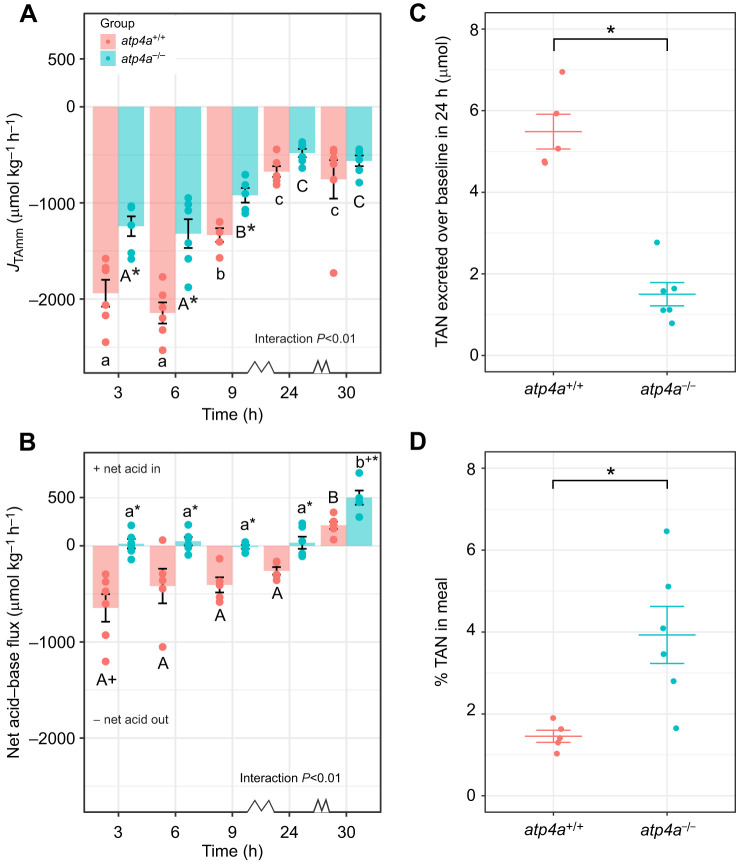
**Ammonia and acid–base fluxes in WT and KO Mexican tetra.** Flux rates (μmol kg^−1^ *M*_b_ h^−1^) of (A) ammonia (*J*_TAmm_) and (B) net acid net acid (H^+^) or base (OH^−^) of *A. mexicanus atp4a*^+/+^ and *atp4a*^−/−^ fed a 5% *M*_b_ ration of bloodworms (*n*=8 per group). Negative values indicate a net base uptake (i.e. acid excretion) and positive values indicate net base excretion (i.e. acid uptake). Different letters indicate significant differences relative to 3 h post-feed excretion. In B, superscript + indicates fluxes significantly different from 0. Data are presented as means±s.e.m. (C) Cumulative total ammonia nitrogen (TAN; μmol) excreted over baseline per group. (D) Percentage of excreted ammonia corresponding to direct absorption from food ammonia, i.e. excluding feed catabolism. Significant differences between genotypes are identified by asterisks (*t*-test or Wilcoxon rank sum test, *P<0.05).

The ammonia excretion rate (*J*_Tamm_) to the water was elevated during the first 24 h post-feed in both genotypes and was 3.65-fold higher in *atp4a*^+/+^ compared with *atp4a*^−/−^ fish following feeding and until 24 h post-feed (Fig. 2A). No significant changes were observed between groups in titratable alkalinity (*J*_Talk_; see Fig. S2). The net acid–base flux was calculated as the difference between *J*_Tamm_ and *J*_TAlk_, with negative values indicative of net base uptake (i.e. acid excretion) and positive values indicative of net base excretion (i.e. acid uptake). The acid–base fluxes showed elevated base uptake levels in *atp4a*^+/+^ animals during the first 24 h and remained unchanged in *atp4a*^−/−^ animals (with net positive values) throughout the same time period. Both groups showed positive flux values (base excretion) at 30 h post-feed, albeit significantly higher in *atp4a*^−/−^ animals (interaction *P*<0.01; Fig. 2B). The bloodworm meal ammonia content averaged 0.077±0.003 µmol TAN. The cumulative amount of ammonia excreted above baseline levels was significantly higher (approximately 5-fold) in *atp4a*^+/+^ in relation to *atp4a*^−/−^ fish (Fig. 2C). The percentage of excreted ammonia corresponding to direct absorption (i.e. excluding catabolism) from the meal (%TAN in meal) was calculated by dividing the excreted TAN over the baseline (µmol) by the amount of ammonia in the meal ingested. This value was significantly higher in *atp4a*^−/−^ animals (*t*_9_=3.181 *P*=0.011; Fig. 2D). The total ammonia concentration measured in the gastric chyme was found to be more than 50% higher in *atp4a*^−/−^ fish (2.55±0.31 mmol l^−1^) relative to *atp4a*^+/+^ fish (1.65±0.17 mmol l^−1^, *t*_27_=2.486, *P*=0.019).

### Reduced SDA magnitude and duration in *atp4a^−/−^ A. mexicanus*

**Fig. 3.**
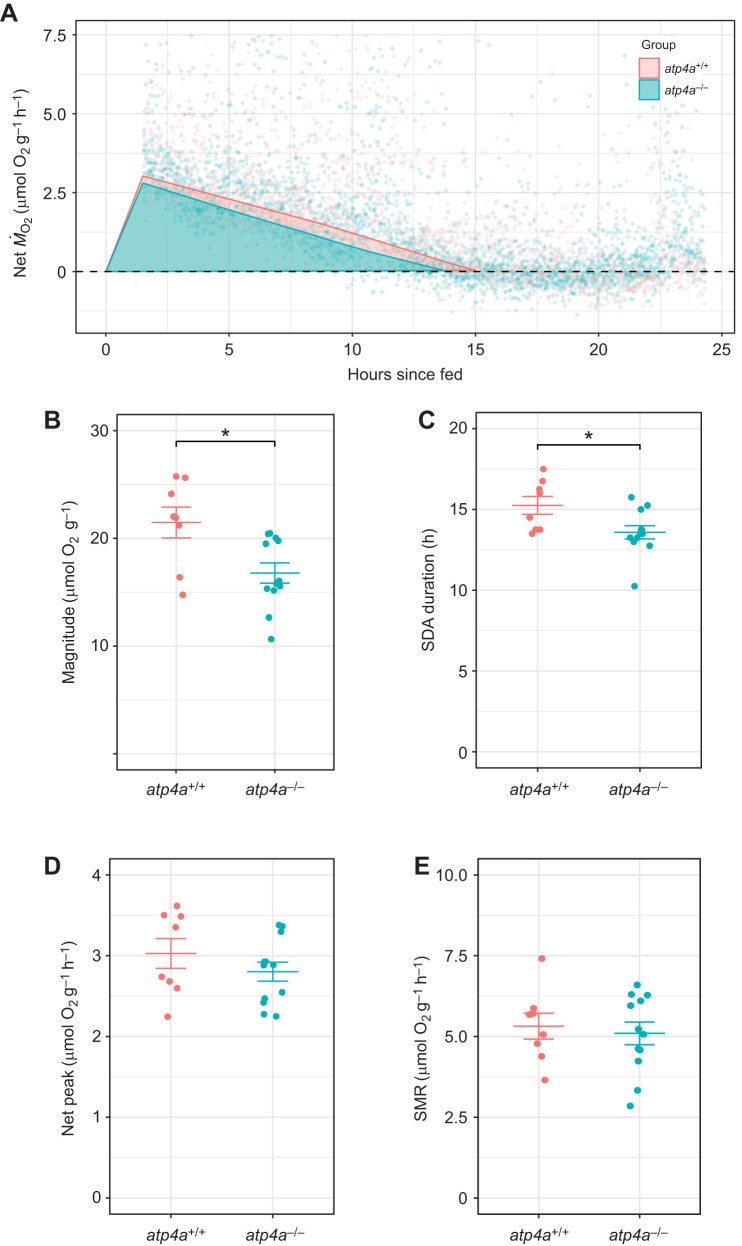
**Change in oxygen consumption rate (*Ṁ*_O_2__) over time in *A. mexicanus* voluntarily fed a 5% *M*_b_ meal of bloodworms (*n*=8 *atp4a*^+/+^ and *n*=12 *atp4a*^−/−^).** In A, points show the recorded *Ṁ*_O_2__ values for all individuals of both groups, while polygons show the average specific dynamic action (SDA) area for each group (i.e. individual SDA values for each time point were averaged for this representation). To ease visualization of the SDA polygons, the *y*-axis was truncated at 7.5 μmol O_2_ g^−1^ h^−1^; hiding 276 individual *Ṁ*_O_2__ values (corresponding to 4.8% of the full dataset). (B) Magnitude, (C) SDA duration, (D) net peak and (E) SMR in both genotypes. Significant differences between genotypes are identified by asterisks (Welsh two sample *t*-test, *P<0.05).

The post-prandial *Ṁ*_O_2__ of *atp4a^+/+^* and *atp4a^−/−^* fish is shown in Fig. 3A. The two genotypes had a similar SMR (*atp4a*^+/+^ 5.42±0.40 µmol O_2_ g^−1^ h^−1^, *atp4a*^−/−^ 5.10±0.35 µmol O_2_ g^−1^ h^−1^, *t*_15.9_=0.418, *P*=0.681), establishing a solid baseline for further comparisons (Fig. 3E). The SDA duration of *atp4a*^−/−^ fish was significantly reduced by 11.0% compared with *atp4a*^+/+^ fish (*atp4a*^+/+^ 15.25±0.55 h, *atp4a*^−/−^ 13.58±0.41 h, *t*_14.1_=2.432, *P*=0.029; Fig. 3C). The net peak of SDA, in contrast, was similar between groups (*atp4a*^+/+^ 3.03±0.18 µmol O_2_ g^−1^ h^−1^, *atp4a*^−/−^ 2.80±0.12 µmol O_2_ g^−1^ h^−1^, *t*_12.6_=1.034, *P*=0.321; Fig. 3D). Finally, SDA magnitude was significantly reduced by 16.8% in *atp4a*^−/−^ animals relatively to *atp4a*^+/+^ fish (*atp4a*^+/+^ 21.47±1.43 µmol O_2_ g^−1^ h^−1^, *atp4a*^−/−^ 16.78±0.94 µmol O_2_ g^−1^ h^−1^, *t*_12.8_=2.745, *P*=0.017; Fig. 3B).

### Lack of stomach acidification does not produce significant changes to appetite

The appetite analysis did not reveal differences between genotypes (Table S2). Likewise, no differences were found in the condition factor (Fulton, *K*) nor in gastric evacuation (Table S2). Finally, no differences were found in any of the genes linked to satiation analyzed in the brain tissues (Fig. S2).

### Gastric acid influences carcass mineral composition

**Fig. 4.**
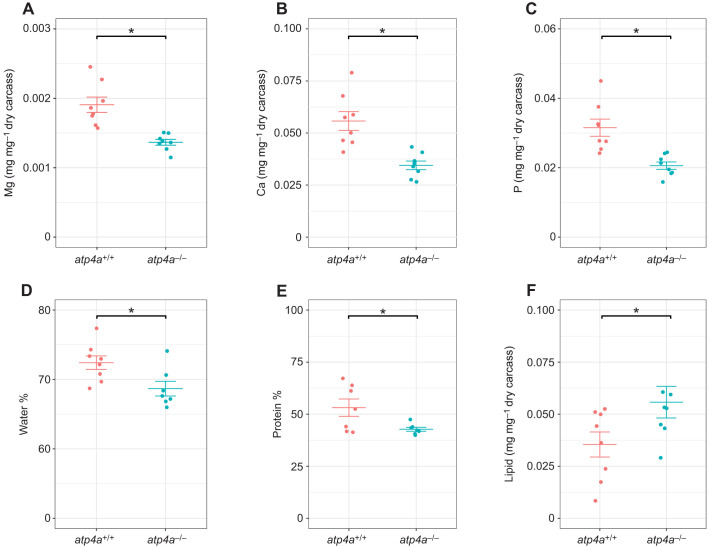
**Carcas analyses of *A. mexicanus*.** Carcass analyses show significantly lower levels of (A) Mg, (B) Ca and (C) P in *atp4a*^−/−^ fish. The percentage of water (D) and protein (E) in the carcass of *atp4a*^−/−^ is significantly reduced, while lipid content (F) is increased. Asterisks indicate a significant difference between genotypes (*t-*test or Wilcoxon rank sum test, *P<0.05).

The carcass composition analyses revealed significantly lower levels of Mg, Ca and P in *atp4a*^−/−^ fish (40%, 53% and 62% reductions, respectively; Fig. 4A–C). The levels of sodium (Na) remained unaltered between genotypes (10.0±0.6 mg g^−1^ of dry carcass *atp4a*^+/+^, 10.0±0.6 mg g^−1^ of dry carcass *atp4a*^−/−^; Fig. S3). The *atp4a*^+/+^ group had a significantly higher carcass moisture content than *atp4a*^−/−^ fish (Fig. 4D). Total carcass protein levels were significantly decreased in *atp4a*^−/−^ fish (Fig. 4E). In contrast, *atp4a*^−/−^ animals had a significantly higher carcass lipid content compared with *atp4a*^+/+^ animals (Fig. 4F).

### Gastric acid knockout decreases digestibility and exacerbates protein faecal loss

**Fig. 5.**
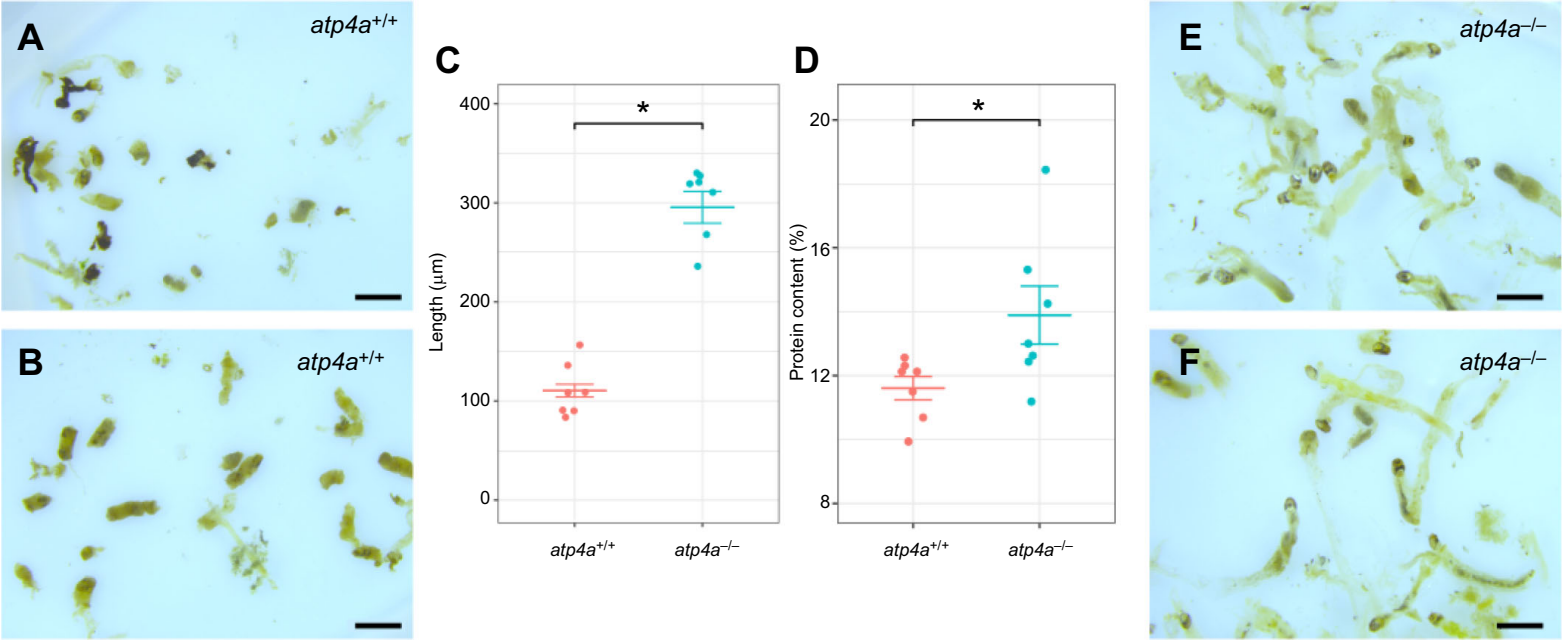
**Changes in digestibility assessed through fecal analysis of animals fed bloodworms.** Visual comparison between *atp4a*^+/+^ (A,B) and *atp4a*^−/−^ (E,F) faeces shows apparent more intact matter in *atp4a*^−/−^ faeces. Scale bars 100 μm. These differences are corroborated by a statistically shorter length of the faeces (C) and significantly higher protein content (D) in faecal matter from *atp4a*^−/−^ fish (*n*=8, *t*-test, *P<0.05).

The faeces of the two groups differed greatly in appearance, with *atp4a*^−/−^ faeces showing more intact bloodworms (Fig. 5E,F) compared with visually more digested faeces excreted by *atp4a*^+/+^ animals (Fig. 5A,B). The length of the digested bloodworms was used as a proxy for the degree of digestion. The overall length of individual faeces was greater in *atp4a*^−/−^ fish (Fig. 5C). The percentage of protein in the faecal matter was also more than 2% higher in *atp4a*^−/−^ animals (Fig. 5D).

### Cholecystokinin levels are negatively impacted by lack of gastric acid

**Fig. 6.**
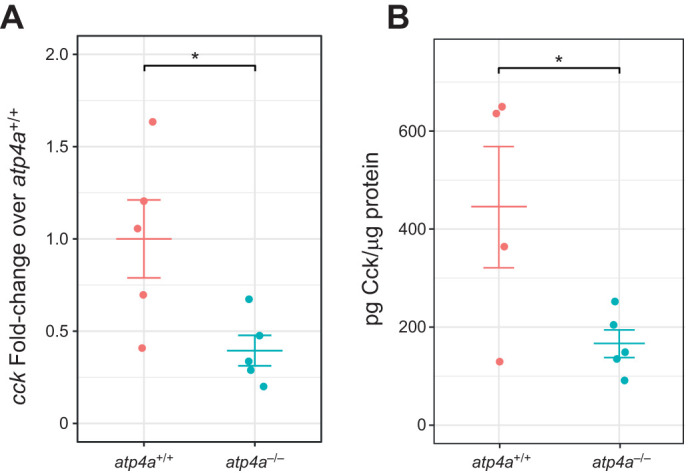
**Changes in anterior intestine *cck* transcript and Cck enzyme levels at 3 h post-feed in *atp4a*^+/+^ and *atp4a*^−/−^ *A. mexicanus*.** (A) *cck* transcript. (B) Cck protein. Asterisks indicate significant differences between genotypes (*t*-test or Wilcoxon rank sum test, *P<0.05).

At 3 h post-feeding, the *cck* mRNA levels in anterior intestine were significantly lower in *atp4a*^−/−^ fish (*t*_12_=2.562, *P*=0.024; Fig. 6A). The Cck peptide concentration in the anterior intestine of *atp4a*^+/+^ fish was 60% lower in *atp4a^−/−^* fish as well (*t*_7_=2.456, *P*=0.044; Fig. 6B).

## DISCUSSION

The inhibition of the gastric proton-pump acid secretion through targeted gene knockout (*atp4a^−/−^*) had profound effects on post-prandial energy utilization and protein, lipid and mineral (P, Mg, Ca) balance but did not affect growth in juvenile *A. mexicanus* during an 8-week growth trial. Impaired protein utilization is corroborated by lower post-prandial ammonia efflux (indicator of protein catabolism), and more intact faeces with a higher protein content (poor protein digestion and/or absorption). The clear effects reported here of decreased protein and increased lipid contents contrast with knockout studies in tetrapods that did not find any changes. Appetite was unaffected, as was brain satiation gene expression. The decreased SDA duration and lower intestinal Cck expression also suggest an increase in gastric emptying rates.

### Impacts on post-prandial metabolic rate

Our results showed a 21.8% decrease in the magnitude of SDA in *atp4a*^−/−^ animals voluntarily fed a bloodworm meal relative to *atp4a*^+/+^ fish. Although the animals in our study did not experience a decrease in growth, the reduction in post-prandial ammonia excretion rates indicate reduced protein catabolism/digestion. Previous studies have reached varying conclusions on the impact of the decreased stomach acidification on SDA. In particular, Moffatt et al. (2022) estimated a decrease of 34.5% in the magnitude of the SDA in Nile tilapia treated with omeprazole. Goodrich et al. (2022a) estimated a value of 45% in magnitude reduction when barramundi (*Lates calcarifer*) were fed acidified diets that were expected to reduce the magnitude of proton pumping by HKA and increase growth efficiency (Lückstädt, 2008). Furthermore, in another study, Goodrich and co-workers (2022b) found that trout fed buffered diets that would lead to an increase in luminal proton pumping to acidify the chyme had an increase of 11% in SDA magnitude. These findings in teleosts contrast with results from studies in snakes that largely failed to detect a discernible impact on SDA (reviewed by Andrade et al., 2005; Henriksen et al., 2015; Nørgaard et al., 2016). The energetic costs of acid secretion and maintenance of the proton pump are thought to be relatively small and to not contribute a significant direct cost in snakes (Wang and Rindom, 2021). To address this discrepancy, in *A. mexicanus* we estimated the metabolic cost of post-prandial gastric acid secretion by calculating acid secretion [from the pH differences between stomach chyme and the bloodworm meal (Ferreira et al., 2025b), bloodworm buffer capacity (Fig. S4) and meal size (5% Mb)], and using published H^+^ secretion:oxygen consumption ratios [5.0 and 2.3 from Kopic et al. (2010) and Bannister (1965), respectively, with the latter taking into consideration inefficiencies because of proton back leak]. The calculated oxygen consumption rates are 0.564 and 1.226 µmol O_2_ g^−1^ *M*_b_. The proton pump acid secretion only accounts for 2.6–5.6% of SDA magnitude of wild-type fish compared with the 21.8% difference in wild-type versus knockout fish. This relatively small direct cost of acid secretion indicates that the impact of the lack of stomach acidification on post-prandial energy expenditure is substantially larger when considering its importance in protein digestion, assimilation and growth. However, it should be kept in mind that there are also likely indirect costs such as mucus and HCO_3_^−^ secretion (alkaline tide and chyme neutralization) that are not considered in these calculations (e.g. Goodrich et al., 2022b).

Additionally, the 11% decrease in the overall duration of the SDA in the *atp4a*^−/−^ fish suggests a faster evacuation time (reduced digestive/absorptive period), which contributes to the overall decrease in SDA magnitude. Previous studies (Couturier et al., 2013; Andersen et al., 2016) positively correlated the buffering capacity of the diet with increased gut transit times, agreeing with the our findings of a shorter SDA time in the absence of stomach acidification. Although we were unable to detect a faster evacuation, this may be explained for technical reasons. The fish were relatively small (∼0.4 g in a species that reaches the 3 g range; Riddle et al., 2021), and thus so were the ingested amounts of feed, and the overall volume capacity of their stomachs that may mask any changes in gastric evacuation due to the lack of necessary resolution in the measurements. In contrast, in a study on much larger Nile tilapia (100 g range), a decrease of 34.4% in the duration of SDA had been previously reported in omeprazole-treated tilapia and related to faster gastric evacuation rates (Moffatt et al., 2022). Future studies should repeat these measurements in larger *A. mexicanus* fed a variety of meal sizes and feed types with a marker to track transit times (Langton, 1977). Importantly, allowing the fish to control intake (by supplying an unlimited amount of food) could dramatically impact transit time. For example, in gastric bypass mice, the treatment animals ate more, and had lower digestibility and faster transit of material through the gut (Liou et al., 2013).

### Cholecystokinin and gastric emptying

An explanation for the poor digestion in *atp4a*^−/−^ fish can be provided by the observed decrease in *cck* mRNA and Cck concentrations in the anterior intestine. The downregulation of *cck* is likely the result of the absence of a feedback mechanism in response to pH changes in the anterior intestine resulting from the entry of an acidic chyme into this portion of the intestinal tract (Goyal et al., 2019). In the *atp4a^−/−^* fish, which lack the capacity to acidify the chyme, the arrival of a non-acidic chyme to the intestine would not trigger a pH-mediated response that leads to the secretion of Cck into the bloodstream and consequent changes in stomach motility and decreased gastric emptying. In this way, this decrease in our Cck data are likely indicative of faster chyme transit from the stomach to the intestine. Importantly, naturally agastric species and herbivores are expected to have faster transit times that are accompanied by more frequent meal ingestion (German, 2009; Jumars, 2000). Thus, the current *atp4a^−/−^* model regarding food intake and satiation fits this pattern as well.

### Digestion, assimilation and growth

Gastric acid provides an important lytic action (Moriarty, 1973) and is essential for the activation of gastric proteases (Kageyama, 2002), and thus a decreased level of protein digestion/metabolism was expected. Indeed, the analyses of the nutrient profile in the carcass of *A. mexicanus* revealed a decreased protein content in *atp4a*^−/−^ fish. These results, combined with an increase in protein content in the faeces consisting of more intact bloodworms, support the importance of HKA in protein digestion and assimilation capacity. In agreement with the lower protein uptake and digestive capacity, we observed significantly lower levels of post-prandial ammonia excretion in *atp4a^−/−^* fish as well. Elevated plasma ammonia levels are produced during digestion from amino acid catabolism in the gut epithelium (Karlsson et al., 2006) and liver (Ip and Chew, 2010).

We also observed that the gastric chyme ammonia concentration was higher in *atp4a*^−/−^ fish. Because the source of this ammonia in the gastric chyme can be accounted for by the ammonia originating in the ingested bloodworm meal, this difference indicates an impairment in the absorption of ammonia from the food by the gut of knockout fish. Our results support a role for the stomach in dietary ammonia absorption, which is consistent with the observation by Jung et al. (2023) in rainbow trout. The mechanism of gastric absorption remains to be defined (Ferreira et al., 2025b). The unabsorbed dietary ammonia in *atp4a*^−/−^ fish is likely excreted in the faeces (Wood and Eom, 2025). However, as faecal ammonia was not measured, its relative contribution to the elevated total nitrogen measured in faeces is unknown.

Notably, our growth data contrast with the reported decrease in growth in Nile tilapia (*Oreochromis niloticus*) treated with the proton pump inhibitor omeprazole (Moffatt et al., 2022). More importantly, because both *atp4a^+/+^* and *atp4a^−/−^* fish grew at similar rates during the 8-week growth trial, it was only when *A. mexicanus* were given a diet supplemented with omeprazole did both groups experience a decreased growth rate. This was accompanied by increased food conversion ratios that are indicative of a reduced ability to convert ingested food into growth. There were no apparent changes to appetite when fish were fed omeprazole-supplemented feed (all fish ingested the entire meal within 3 h, similarly to observations with untreated feed). This effect persisted for the first few weeks after the return to a non-treated diet. These findings point to possible off-target effects of omeprazole that inhibit growth independently of acid secretion, as the *atp4a^−/−^* fish are achlorhydric already. In general, omeprazole is noted as a specific HKA inhibitor because of the need for acidification to activate the inhibitor (Huttunen et al., 2011), and there are relatively few side effects when prescribed to treat gastroesophageal reflux disease (GERD), gastric ulcers and gastric reflux (Schubert, 2019). However, off-target effects have been reported, although not without some controversy (Cartee and Wang, 2020; Perry et al., 2020). Of note, previous experiments in sea urchins (*Strongylocentrotus purpuratus*) by Stumpp et al. (2015) showed an effect of omeprazole on gastric alkalization in echinoderm tornaria larvae, an organism that lacks the HKA altogether (Okamura et al., 2003), further corroborating the hypothesis of the existence of secondary targets of this drug. This is an important finding, as it reveals an off-target effect of omeprazole, raising important questions on possible secondary effects of this widely used drug.

It is important to consider that a longer growth trial period may reveal long-term impacts in growth rates that were not detected in the present study (e.g. Cohen-Rothschild et al., 2024). Notably, the *atp4a^−/−^* fish were significantly smaller at the end of the recovery period. However, these results could be, among other hypotheses, the result of the impact of the omeprazole diet on the gut microbiome and digestive capacity that may differentially affect the two genotypes. These findings pose important questions regarding the modulation of the gut microbiome by omeprazole in the presence and absence of gastric acid, opening the way to further research.

The *atp4a*^−/−^ fish had a higher lipid content, which is a novel finding that has not been reported in any previous study of knockout or knockdown of HKA (Judd et al., 2005; Moffatt et al., 2022; Zhao et al., 2010). The analysis of the mineral content of the carcasses also revealed a decrease in phosphorus content. The phosphorus requirements of fish are met mainly through diet (NRC, 2011). Notably, phosphorus is found in fish meal as hydroxyapatite (an insoluble Ca–P complex), and increased availability has been linked to acid hydrolysis (Albrektsen et al., 2018; NRC, 2011; Sugiura et al., 2006). This agrees with our data, whereby gastric acid secretion plays an important role in the solubilization of phosphate from the diet. In contrast, aquaculture feed companies often improve digestibility by supplementing diets with inorganic phosphorous salts (Hua and Bureau, 2006). Significantly, previous studies (Abuduli et al., 2016; Chun et al., 2016; Wong, 2022; Zhang et al., 2023) have also established the importance of phosphorus in the regulation of body lipid content, through regulation of lipid biosynthesis and oxidation pathways. In this way, the increase in body fat observed in *atp4a^−/−^* fish in the present study is likely related to phosphorus malabsorption. In addition, the presence of phosphorus is important for calcium regulation (Taylor and Bushinsky, 2009). Calcium and phosphorus are of critical importance in the development and maintenance of skeletal function, among other physiological functions related to acid–base homeostasis (Fontagné et al., 2009; Lall and Kaushik, 2021; Zimmer et al., 2019). Indeed, our results show a decrease in carcass calcium content in *atp4a*^−/−^ fish as well. Future research should aim to determine changes in bone density and to explore possible changes in the skeleton at earlier stages of development.

Our findings are consistent with reports of hypomagnesaemia under pharmacological knockdown of the proton pump with proton pump inhibitor administration (Jaworek, 2020; Judd et al., 2005; Schubert, 2019; Zhao et al., 2010). Magnesium is a divalent cation that plays an essential role in the physiological processes of protein synthesis, cell replication and energy metabolism, and, in bony fishes, 50–70% or more is stored in skeletal tissues and scales (Bijvelds et al., 1998; Lall and Kaushik, 2021) in contrast to 99% of calcium and 80% of phosphorous (FAO, 1980). The magnesium requirement for fish is mainly achieved through diet, and several mechanisms for intestinal transcellular magnesium uptake have been identified in teleosts (*slc41a1*, *trpm6*; Arjona et al., 2019; Kodzhahinchev et al., 2017). Further studies are needed to investigate the causes of the significant decrease in Mg body content in *atp4a^−/−^* fish and possible links to decreased transcellular Mg^2+^ transport.

### Alkaline tide

When acid is secreted into the stomach lumen, the base that is generated makes its way into the bloodstream as the alkaline tide (Brunton, 1933; Niv and Fraser, 2002). The small size of the fish precluded the possibility of measuring the alkaline tide directly in the blood, but it might be observable as an increase in the whole-animal base flux into the water (Wood, 2019). However, the predicted post-prandial alkaline tide in the blood did not translate to an increase in base efflux in *atp4a^+/+^* animals. Instead, within the first 24 h after feeding, net acid excretion was observed in *atp4a*^+/+^ fish, corresponding to base uptake, whereas the net base excretion was not different from zero in *atp4a*^−/−^ animals. This is likely due to the nature of the meal and the small ration size fed to the animals (5% *M*_b_ wet bloodworms, ∼1% *M*_b_ dry bloodworms), which may be insufficient to produce substantial base excretion into the water (Wood, 2019). Our findings are comparable to those obtained by Cooper and Wilson (2008), who showed a negligible net base excretion in rainbow trout fed a 1% *M*_b_ meal of pellets, which contrasts with observations of a substantial flux in rainbow trout fed a larger 5% *M*_b_ meal (Bucking and Wood, 2008) and in barramundi fed a 2.5% *M*_b_ ration (Goodrich et al., 2022b). Another relevant component at play in the interpretation of these data is the large increase in ammonia excretion. Thus, although these data do not support the existence of a post-prandial base excretion in *A. mexicanus* fed a 5% *M*_b_ wet bloodworm meal, the significant decrease in ammonia excretion in *atp4a*^+/+^ is likely driving the differences seen in net acid–base flux between the two groups.

### Conclusions

Our study addresses long-standing physiological questions in gastrointestinal physiology with resource to a genetic knockout of the gastric proton pump. This approach reduces confounding factors that may arise from indirect gastric modulation methods. Taken together, the lower protein digestibility, lower ammonia metabolism and possible changes to gut transit times further support the changes in SDA in HKA-null animals reported in this study. In previous studies of HKA modulation (Secor, 2009; Moffatt et al., 2022; Goodrich et al., 2022a,b), and from our own findings, it is clear that the HKA importance transcends the direct cost of acidification and is likely related to changes in nutrient assimilation caused by the absence of acid (Goodrich et al., 2024). This study is the first to report a significant depletion in ammonia excretion rates following inhibition/ablation of HKA in vertebrates. However, conclusions should be made cautiously because the animals in this study were fed a high protein diet and the same trend may not be noticed with different food sources. We have linked a decrease in acidification to a reduced SDA, in agreement with previous studies, but no decrease in growth efficiency. We expect that the knockout of gastric acid in an obligate carnivore species would produce a notable effect in growth rates as it is clear the absence of gastric acidification precludes an efficient digestion of proteins. We have established a direct link between gastric acid secretion and magnesium, calcium and phosphate balance in *A. mexicanus*. This new knockout line offers a good model for the study of mineral bone diseases, representing an advantage over currently used fish models that are agastric (Lleras-Forero et al., 2020) because it enables a direct comparison of different bone phenotypes within the same species. Lastly, these findings offer new insights into the importance of the stomach as an evolutionary innovation in gnathostomes, while opening the way for a better understanding of the physiological pressures and demands of the secondary loss events of this organ in numerous vertebrate clades.

## Supplementary Material

10.1242/jexbio.251599_sup1 Supplementary information
