# Supplementary material for: Lack of gastric acidification reduces postprandial energy expenditure and protein digestion but not growth in Astyanax mexicanus
Source: J Exp Biol. 2026 Apr 30;229(9):jeb251599. doi: 10.1242/jeb.251599 (PMC13200723; doi:10.1242/jeb.251599)
Supplement: Supplementary information [file jexbio-229-251599-s1.pdf]

Table S1. Growth trial pellet formulation (Tropical micro pellets, Hikari)

MICRO PELLETS®

INGREDIENTS:

Fish meal, krill meal, soybean meal, flaked corn, cuttlefish meal, brewers dried yeast, wheat germ meal, wheat starch, fish oil, powdered cellulose, hydrolyzed vegetable sucrose polyesters, dried seaweed meal, lecithin, DL-methionine, garlic, spirulina, astaxanthin, choline chloride, vitamin E supplement, L-ascorbyl-2-polyphosphate (stabilized vitamin C), inositol, d-calcium pantothenate, riboflavin, vitamin A supplement, thiamine mononitrate, pyridoxine hydrochloride, niacin, folic acid, vitamin D3 supplement, biotin, vitamin B12 supplement, P-aminobenzoic acid, disodium phosphate, salt, ferrous sulfate, magnesium sulfate, zinc sulfate, manganese sulfate, copper sulfate, calcium iodate, red 3, yellow 5 (artificial colors).

GUARANTEED ANALYSIS:

Crude Protein	Crude Fat	Crude Fiber	Moisture	Ash
min. 43.0%	min. 7.0%	max. 7.0%	max. 10.0%	max. 17.0%
Phosphorus	Vitamin A	Vitamin D3	Vitamin E	Ascorbic Acid
min. 1.1%	min. 16,000 IU/kg	min. 2,400 IU/kg	min. 960 IU/kg	min. 360 mg/kg

Journal of Experimental Biology • Supplementary information

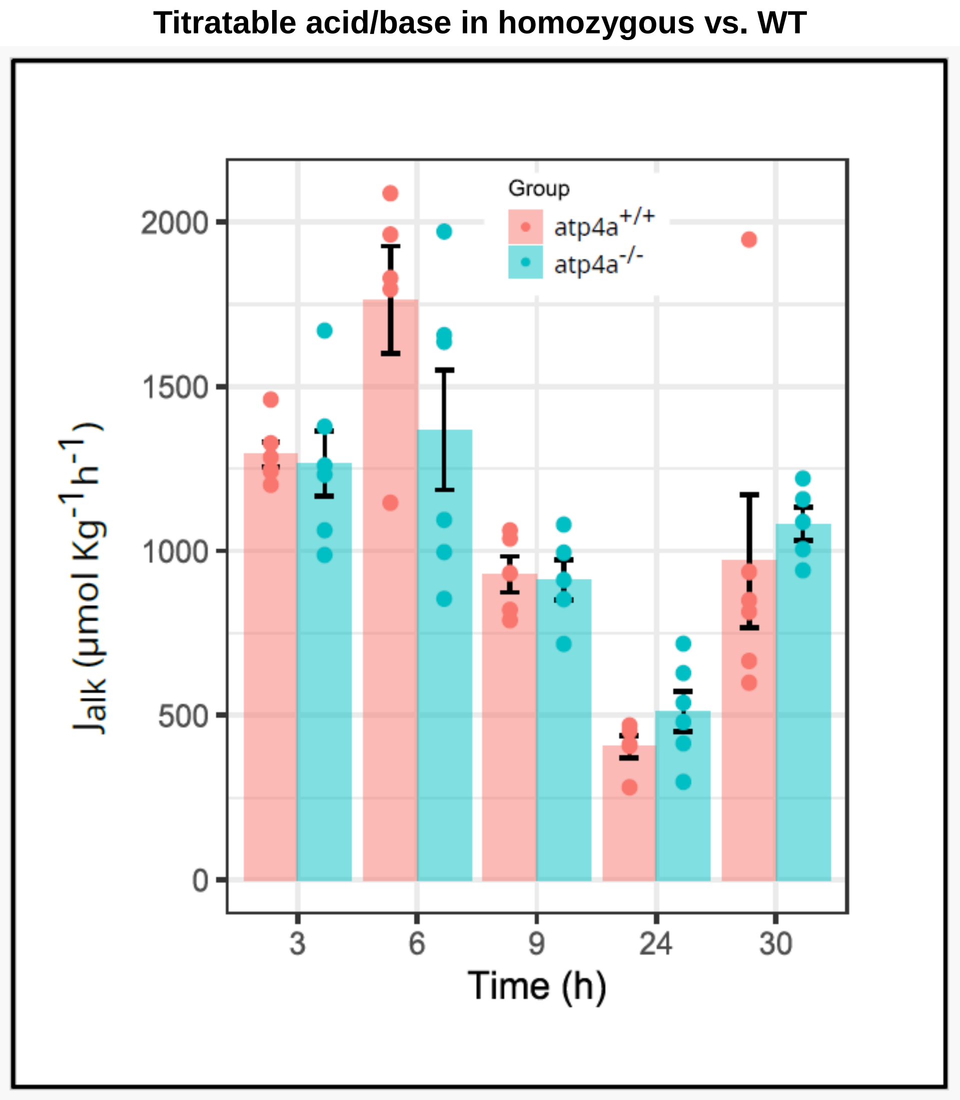

Fig. S1. Water titratable alkalinity of *A. mexicanus atp4a*$^{+/+}$ and *atp4a*$^{-/-}$ fed a 5% BM ration of bloodworms (n = 8 per group). Data are presented as mean ± SEM.

Journal of Experimental Biology • Supplementary information

Brain transcriptional analysis

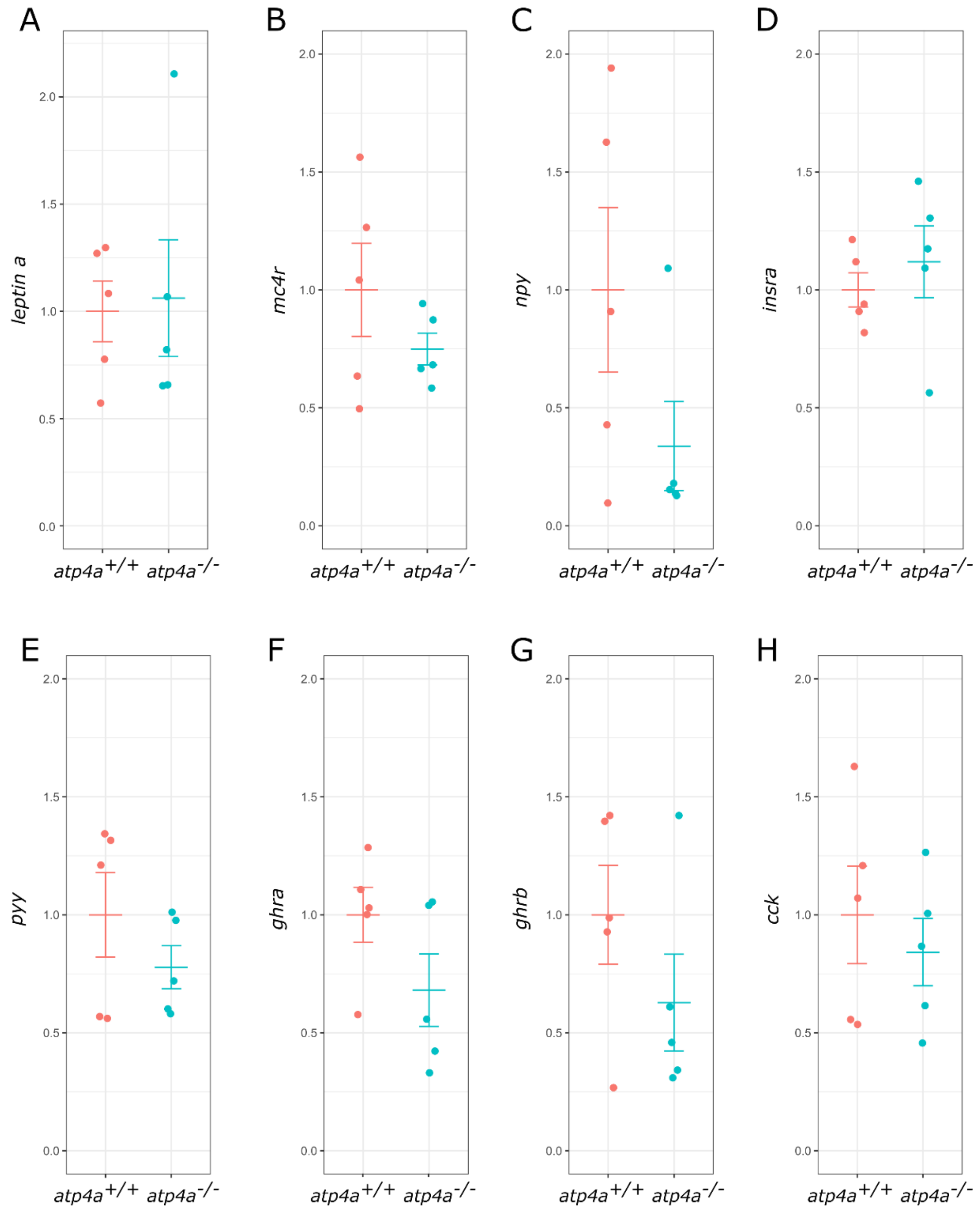

Fig. S2. Analysis of transcriptional changes of selected genes related to satiation and growth in brain tissue. The brain mRNA levels for *leptin a* (**A**), *mc4r* (**B**), *npy* (**C**), *insra* (**D**), *pyy* (**E**), *ghra* (**F**), *ghrb* (**G**) and *cck* (**H**) remained unaltered between groups at 3h post-feed. Gene expression presented as fold over $atp4a^{+/+}$fish. t-test $p < 0.05$.

Journal of Experimental Biology • Supplementary information

Carcass analysis

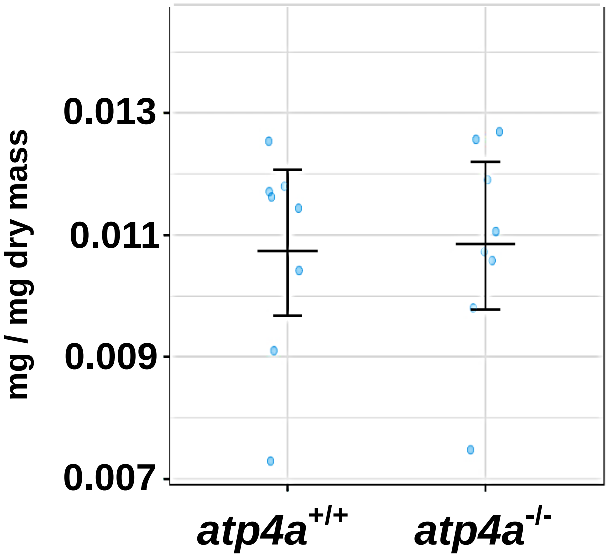

Fig. S3. Carcass sodium content (mg Na /mg dry mass). Groups compared by t-test. No significant differences.

Journal of Experimental Biology • Supplementary information

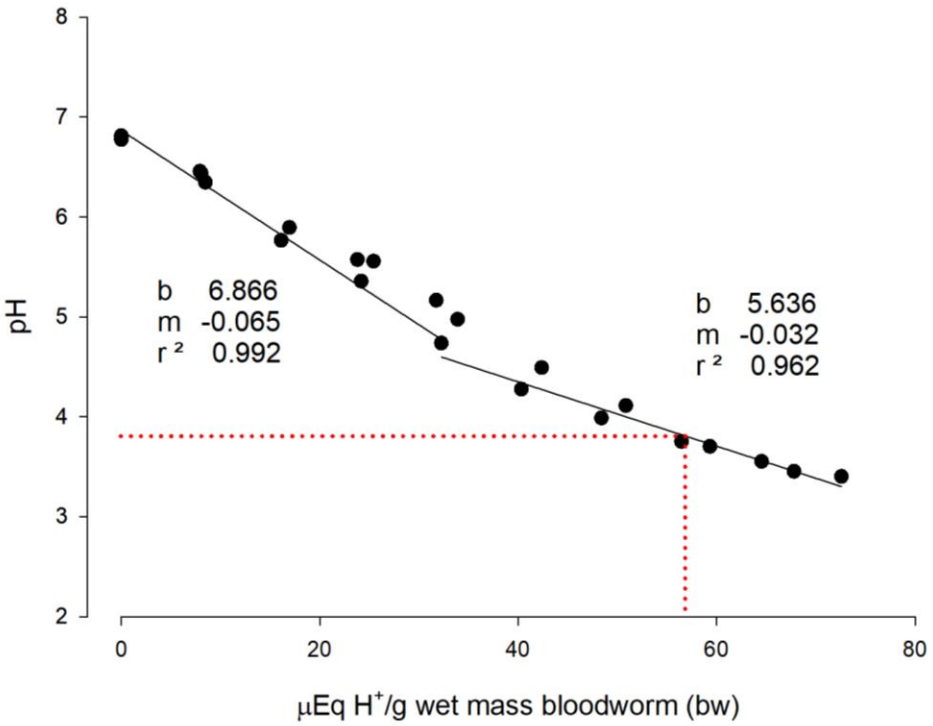

pH Range 6.8-5.0
Gastric acid secreted = [(pHbw-pHst) - 6.866] / -0.065
pH Range < 5.0
Gastric acid secreted = [(pHbw-pHst) - 5.636] / -0.032

Fig. S4. Titration curve showing the net acid secretion required for the bloodworm meal to reach pH 3.8 (red dotted line, value based on Ferreira et al., 2024b).

Journal of Experimental Biology • Supplementary information

Appetite

Table S2. Comparison of appetite [worms consumed (mg/K)], Fulton's condition factor (K), and gastric evacuation [gastric content (wet mass)/ ration (wet mass)] between *atp4a*$^{+/+}$ and *atp4a*$^{-/-}$ fish. Welsh t-test.

	worms consumed (mg/K)	K	Stomach Contents
atp4a$^{+/+}$	4.38 ± 0.21	2.09 ± 0.06	0.18 ± 0.02
atp4a$^{-/-}$	4.66 ± 0.19	1.97 ± 0.07	0.18 ± 0.03
t_{df}	$t_{8.5}$= 1.01	$t_{12.7}$= 1.65	$T_{13.9}$= 0.02
p-value	0.34	0.12	0.98

Journal of Experimental Biology • Supplementary information

Supplementary Materials and Methods

Lipid extraction and determination

Briefly, 10-20 mg of ground carcass was weighed into 2 ml tubes containing 1.8 mL of chloroform and methanol mixture (2:1) and incubated overnight at room temperature with agitation in a fume-hood. Following incubation, 0.2mL of 0.9M NaCl was added and thoroughly mixed by vortexing and incubated for 6h at room temperature followed by a centrifugation at 2.5k x g for 5 min. After centrifugation, the chloroform phase was transferred into a new pre-weighed (using an ultramicrobalance SE2, Sartorius) 1.5mL tube. The tubes were left open in a fumehood at room temperature for 72h until all the chloroform had evaporated. Following this, the tubes were reweighed using the ultramicrobalance and the total lipid was calculated and expressed as a percentage of the dry mass.

Respirometry

The setup consisted of a water bath (approx. 43L) equipped with a portable UV filter and aeration, where four custom-built respirometers were placed. The respirometers were made of clear PVC pipe (inner diameter = 2cm; length = 6 cm) with a total volume of 24 mL including the recirculation tubing. Water temperature during experiments ranged between 21.8 and 23.8°C ($\bar{x}$ = 22.3°C), using an aquarium heater connected to a temperature control system (ITC-306T, Inkbird Tech CL, China). Each respirometer was rinsed with 70% alcohol and left to dry between every run to minimize background oxygen consumption. The respirometers were equipped with O2 probes (OXFLOW-HS; PyroScience GmbH, Aachen, Germany) connected to a PyroScience Firesting O2 sensor (FSO2-C4; PyroScience GmbH). Water temperature was monitored with a temperature probe (TDIP15; PyroScience GmbH) placed within the recirculation loop of one of the chambers. In-chamber oxygen concentration and temperature were measured every second. The flow of water within the chamber was maintained at a level that allowed for accurate O2 measurements but without causing turbulence or disturbance of the fish inside (using 5V pumps, model AD20P-0510A, Shenzhen Giant Electric Tech Inc). One flush pump (same model) was used to flush all four respirometers. The custom-built flush controller was set to perform cycles of 3 min measurement and 2 min flush. The first 60 s of the measurement data were discarded (wait phase). Background oxygen consumption was measured both before adding the animals and after their removal (x-y cycles), to correct for any microbial consumption of O2 that could otherwise have confounded the measurements.

$\dot{M}_{O_2}$ *and SDA calculations*

The $\dot{M}_{O_2}$ for each cycle was determined using the R package pyroresp (Flávio, 2025), in R v4.4.1 (R Core Team, 2024). The recorded O2 values (hPa) were converted to µmol O2 L-1 h-1 using the respirometry R package (Birk, 2024). Changes in background respiration were linearly modeled over time to correct recorded oxygen readings. The corrected O2 readings were used to determine

the slope and R2 of the lines of best fit for each cycle. Cycles with an R2 > 0.9 were considered valid for the determination of $\dot{M}_{O_2}$. SMR was determined by calculating the quantile 0.2 for the pre-feeding $\dot{M}_{O_2}$ values of each animal (Chabot et al., 2016a). SDA was determined using a modified version of the functions provided by Chabot et al. (2016b). Specifically, the function rqss() from the quantreg R package (Koenker et al., 2018) was used to fit an additive quantile regression model to the postprandial $\dot{M}_{O_2}$ data for each animal, with lambda = 24, and tau = 0.2. Trials with fasted animals showed that it takes approximately 1 h for the fish to reduce its O2 consumption to resting levels after being placed in the chamber. As such, data collected during the first 1.5 h post-feeding were not used for SDA determination. Fitted values were predicted for every 15 min interval after the discarded period. The fitted values were used to determine the duration, peak in net $\dot{M}_{O_2}$, and magnitude (the latter using the function trap.rule() from the R package Hmisc; Harrell Jr et al., 2019).

References

Chabot, D., Koenker, R., & Farrell, A. (2016a). The measurement of specific dynamic action in fishes. Journal of Fish Biology, 88 (1), 152–172.

Chabot, D., Steffensen, F., & Farrell, A. (2016b). The determination of standard metabolic rate in fishes. Journal of Fish Biology, 88 (1), 81−121.

Flávio, H. (2025). pyroresp: Analyse respirometry data captured by firesting loggers. https://github.com/hugomflavio/pyroresp

Harrell Jr, F. E., Harrell, J., & Maintainer, F. E. (2019). Package hmisc. CRAN2018, 2019, 235–236.

Koenker, R., Portnoy, S., Ng, P. T., Zeileis, A., Grosjean, P., & Ripley, B. D. (2018). Package ‘quantreg.
